# Astragaloside IV Improves Metabolic Syndrome and Endothelium Dysfunction in Fructose-Fed Rats

**DOI:** 10.3390/molecules16053896

**Published:** 2011-05-10

**Authors:** Ning Zhang, Xu-Hui Wang, Shi-Long Mao, Feng Zhao

**Affiliations:** 1 Department of Pharmacy, Xuhui District Central Hospital, Shanghai 200031, China; 2 Department of Pharmacy, Putuo District Central Hospital, Shanghai 200062, China; 3 Department of Cardiothoracic Surgery, Changhai Hospital, Second Military Medical University, Shanghai 200433, China

**Keywords:** astragaloside IV, fructose, hypertension, metabolic syndrome, vessel function

## Abstract

The prevalence of metabolic syndrome has increased in modern society and the condition is proving to be a common precursor of cardiovascular disease. The aim of the present study was to investigate whether astragaloside IV, a major active constituent of *Astragalus membranaceus* (Fisch) Bge*.*, is able to prevent the development of hypertension and endothelial dysfunction in fructose-fed rats. Rats were fed with 10% fructose in their drinking water for 8 weeks. From the beginning of week 5, two groups of fructose-fed rats were treated with 0.5 or 2 mg/kg, i.p., astragaloside IV. Another group of fructose-fed rats, injected with the same volume of vehicle (dimethylsulfoxide, DMSO) from week 5, served as the control group. At the end of the treatment period, blood pressure, blood glucose, glucose tolerance, blood insulin and lipids were determined. In addition, in vitro experiments were conducted at the end of the eight week treatment period to evaluate endothelium-dependent aortic vasorelaxation, as well as myocardial and aortic tissue levels of nitrate and nitrite (NOx) and cGMP. Fructose-fed rats developed clustering signs of metabolic syndrome, such as increased bodyweight, mild hypertension, hyperinsulinaemia, hypertriglyceridaemia, impaired glucose tolerance and impaired endothelium-dependent vasorelaxation. Administration of astragaloside IV reduced blood pressure and triglyceride levels in fructose-fed rats and high dose of astragaloside IV also improved glucose tolerance and endothelium-dependent vasorelaxation. The astragaloside IV-induced improvement in vasorelaxation was associated with increased levels of aortic NOx and cGMP and was abrogated by blockade of nitric oxide synthase with NG-nitro-l-arginine methyl ester (l-NAME). On the basis of its favourable effects on lipid metabolism, endothelium-dependent vasorelaxation and the nitric oxide–cGMP-related pathway, astragaloside IV may be useful in ameliorating food-induced metabolic syndrome.

## 1. Introduction

**Figure 1:**
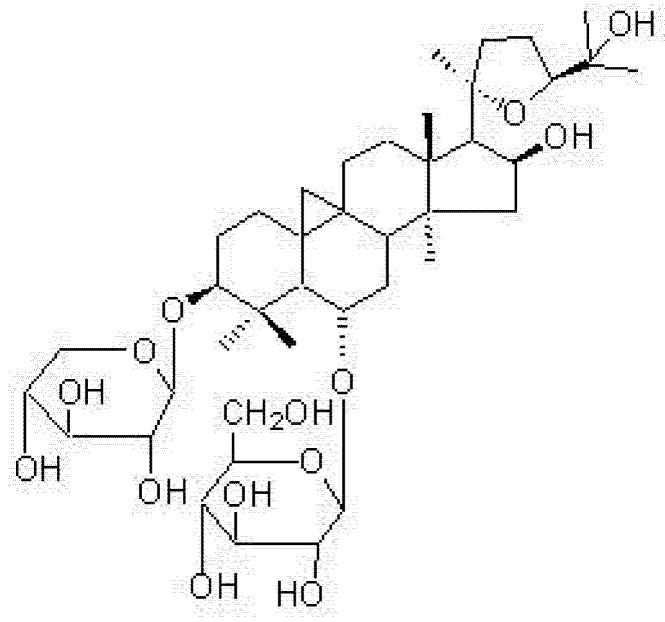
Chemical structure of astragaloside IV.

The prevalence of metabolic syndrome has increased in modern society and the condition is proving to be a common precursor of cardiovascular disease and Type 2 diabetes mellitus. The prevention and treatment of metabolic syndrome are important in reducing cardiovascular morbidity and mortality. Astragaloside IV (Figure 1) is commonly used in the treatment of many disorders, including cardiovascular diseases. Astragaloside IV is the major active component extracted from the traditional Chinese medicine *Astragalus membranaceus* (Fisch) Bge. Previous studies have shown that astragaloside IV can protect the myocardium and central nervous system against ischaemic injury [1,2,3,4]. It also produces vasorelaxation, mainly through the nitric oxide (NO)–cGMP pathway [5,6]. Because metabolic syndrome is frequently accompanied by endothelial dysfunction and increased blood pressure, the effects of astragaloside IV on vessel function may be of some value in preventing these changes in metabolic syndrome.

It has been reported recently that astragaloside IV can increase insulin-induced preadipocyte differentiation, improve high glucose-induced insulin resistance in adipocytes and prevent tumour necrosis factor (TNF)-α-induced apoptosis in endothelial cells *in vitro* [7]. However, it is not clear whether astragaloside IV can exert any beneficial effects on metabolic syndrome *in vivo* and, if so, the mechanisms involved. Rat fed fructose in diet is a suitable model for non obese rats with some aspects of the metabolic syndrome such as hypertension, hyperinsulinemia and hypertriglyceridemia [8]. In the present study, we used fructose-fed rats as a model of metabolic syndrome to investigate whether chronic administration of astragaloside IV can prevent the development of metabolic syndrome and vessel dysfunction in these rats [9,10,11].

## 2. Results and Discussion

### 2.1. Bodyweight and Blood Pressure

**Figure 2:**
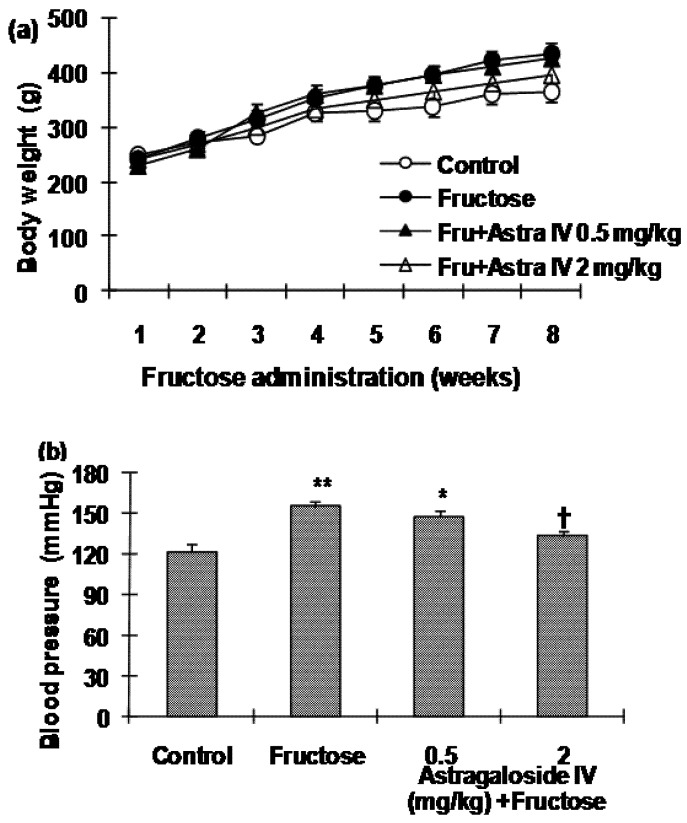
**(a)** Changes in bodyweight over the course of the experimental period and **(b) **systolic blood pressure (SBP) in normal control and fructose-fed rats, with and without astragaloside IV treatment from Week 5. Data are the mean ± SEM (n = 12). *P < 0.05, **P < 0.01 compared with age-matched controls; †P < 0.05 compared with fructose-fed rats.

Figure 2a shows bodyweight gains in the different groups over the eight week experimental period. There was a tendency for body weight to be greater in fructose-fed rats compared with the normal control rats, but the difference did not reach statistical significance. Similarly, there was a tendency for food intake to be decreased in fructose-fed rats, but again the differences failed to reach statistical significance. Blood pressure in fructose-fed rats was significantly higher at the end of the 8-week treatment period compared with normal control rats. Administration of 2 mg/kg astragaloside IV to fructose-fed rats from week 5 reduced the fructose-induced increase in blood pressure. Although there was a tendency for 0.5 mg/kg astragaloside IV treatment of fructose-fed rats to reduce blood pressure, the difference did not reach statistical significance (Figure 2b).

### 2.2. Blood Glucose, Lipids and Glucose Tolerance

**Figure 3:**
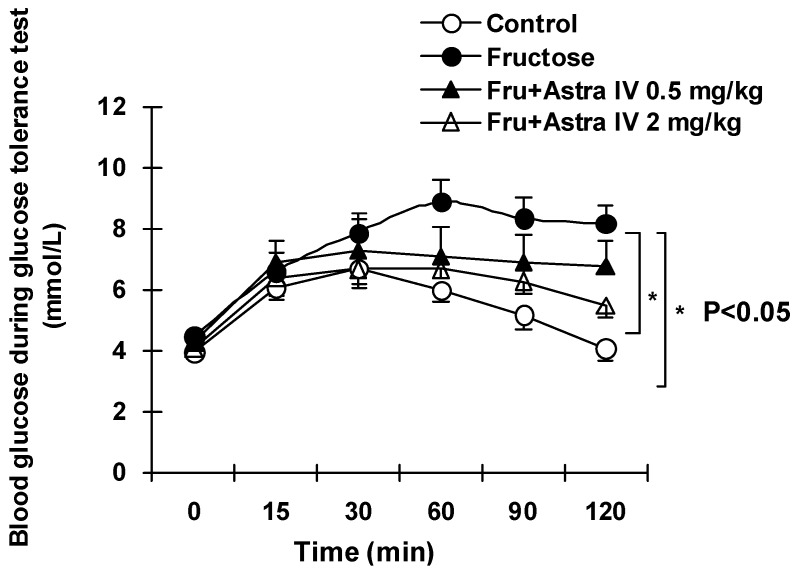
Results of the glucose tolerance test in normal control and fructose-fed rats, with and without astragaloside IV treatment from week 5. Fructose feeding for eight weeks induced significant glucose intolerance, manifested as higher glucose levels and a delayed glucose peak after glucose loading. Data are the mean ± SEM (n = 12). *P < 0.05 compared with fructose feeding alone.

As indicated in Table 1, fructose feeding had a significant impact on insulin and blood lipids. Although fasting blood glucose was normal, insulin concentrations in fructose-fed rats increased significantly compared with concentrations in the normal control group. Treatment of fructose-fed rats with astragaloside IV dose-dependently ameliorated the changes in insulin and TG (Table 1). In the GTT, the increases in blood glucose concentrations after glucose challenge were significantly higher in fructose-fed rats than in the normal control rats; this response was ameliorated in fructose-fed rats following treatment with 2 mg/kg astragaloside IV (Figure 3; Table 1).

**Table 1:** Characteristics of normal and fructose-fed rats, with and without astragaloside IV treatment from week 5.

	Control	Fructose	Fructose + 0.5 mg/kg astragaloside IV	Fructose + 2 mg/kg astragaloside IV
Fasting glucose (mmol/L)	4.0 ± 0.6	4.5 ± 0.8	4.3 ± 0.7	4.1 ± 0.9
Serum insulin (ng/mL)	1.22 ± 0.25	3.95 ± 0.32**	2.77 ± 0.20†	2.13 ± 0.20†
Triglyceride (mmol/L)	1.18 ± 0.13	2.53 ± 0.18**	1.93 ± 0.17†	1.23 ± 0.12††
Total cholesterol (mmol/L)	1.91 ± 0.04	2.05 ± 0.06	1.99 ± 0.07	1.95 ± 0.10
LDL-C (mmol/L)	0.42 ± 0.03	0.44 ± 0.06	0.45 ± 0.04	0.41 ± 0.06
HDL-C (mmol/L)	1.17 ± 0.06	1.30 ± 0.11	1.24 ± 0.10	1.22 ± 0.13

Data are the mean ± SEM (n = 12). *P < 0.05, **P < 0.01 compared with age-matched controls; †P < 0.05, ††P < 0.01 compared with fructose-fed rats; NO_x_, nitrate and nitrite. LDL-C, low-density lipoprotein–cholesterol; HDL-C, high-density lipoprotein–cholesterol.

### 2.3. Vessel Relaxation

**Figure 4:**
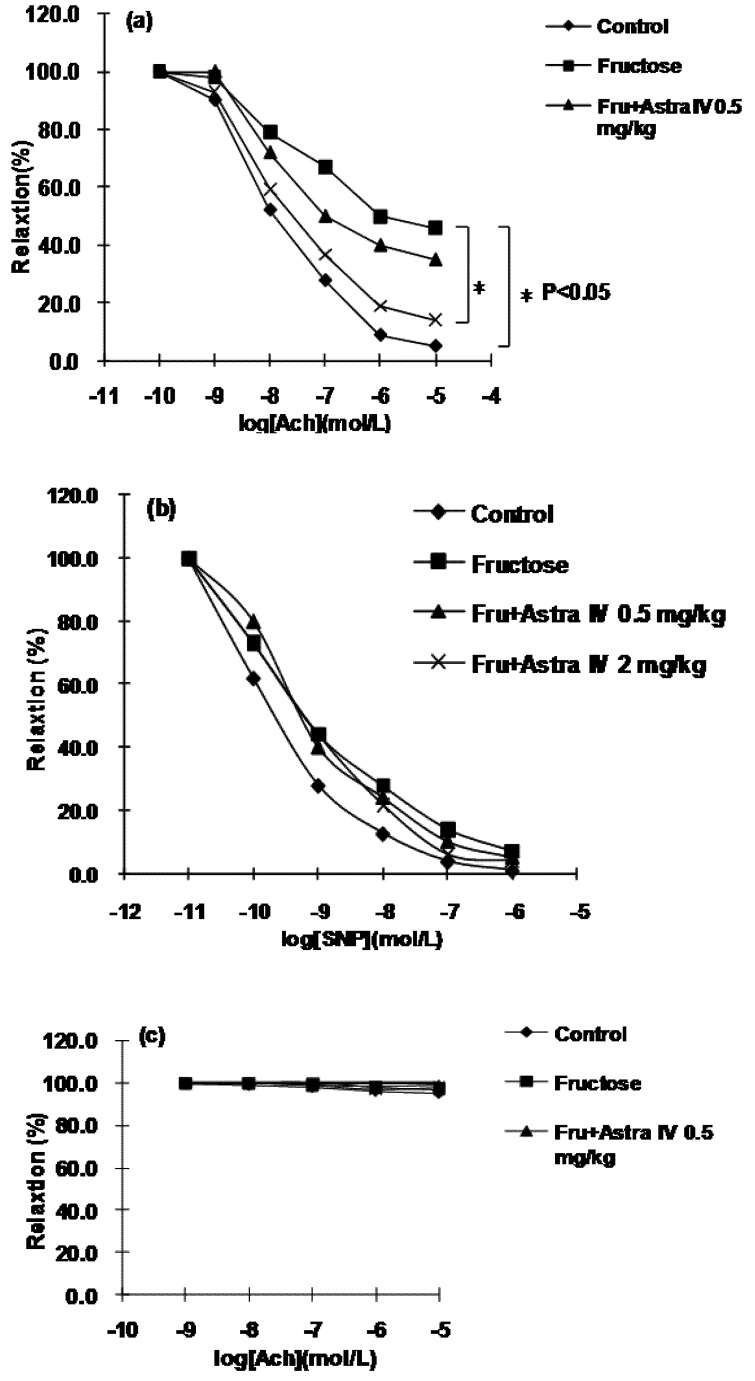
**(a)** Endothelium-dependent vasorelaxation of aortic rings from normal control and fructose-fed rats, with and without astragaloside IV treatment from Week 5, in response to acetylcholine (ACh), **(b)** endothelium-independent vasorelaxation in response to sodium nitroprusside (SNP) and **(c) **effects of inhibition of nitric oxide synthase (NOS) by 10 μmol/L Nω-nitro-l-arginine methyl ester (l-NAME) on endothelium-dependent vasorelaxation in response to ACh. Fructose feeding significantly impaired endothelium-dependent vasorelaxation, but had no effect on endothelium-independent vasorelaxation. Treatment of rats with astragaloside IV restored the endothelium-dependent relaxation and this effect was abolished by NOS inhibition with l-NAME. Data are the mean ± SEM (n = 12). *P < 0.05 compared with age-matched controls. PE, phenylephrine.

As shown in Figure 4a, fructose feeding for eight weeks significantly impaired endothelium-dependent vasorelaxation, as indicated by the reduced relaxation response of aortic rings from fructose-fed rats to ACh. However, there was no obvious change in endothelium-independent vasorelaxation (*i.e.*, in response to SNP) of endothelium-denued rings from fructose-fed rats (Figure 4b). 

In endothelium-intact rings from astragaloside IV-treated fructose-fed rats, the endothelium-dependent relaxation in response to ACh was restored, but these actions of astragaloside IV were abolished by inhibition of NOS with 10 μmol/L l-NAME (Figure 4c). These results indicate that NO is involved in the improvement in vessel function after treatment of rats with astragaloside IV.

### 2.4. Aortic Levels of NOx and cGMP

**Figure 5:**
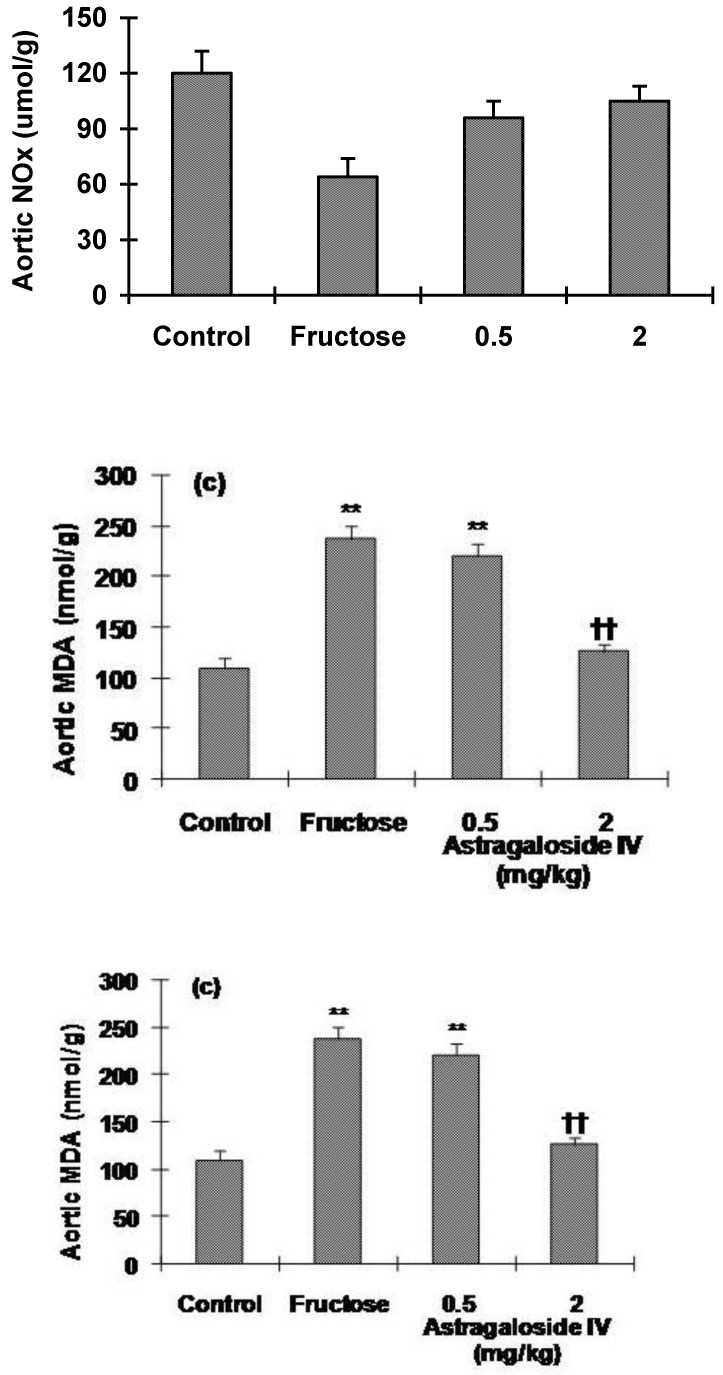
**(a)** Nitrate and nitrite (NO_x_), **(b)** cGMP and **(c) **malondialdehyde (MDA) levels in the aorta of normal control and fructose-fed rats, with and without astragaloside IV treatment from Week 5. Data are the mean ± SEM (n = 12). *P < 0.05, **P < 0.01 compared with age-matched controls; ^†^P < 0.05, ^††^P < 0.01 compared with fructose-fed rats.

As shown in Figure 5a, aortic NO_x_ content was decreased significantly in fructose-fed rats, whereas astragaloside IV treatment of fructose-fed rats increased NO_x_ content. Similar results were seen for aortic cGMP, with the decreased levels in fructose-fed rats ameliorated in rats treated with 2 mg/kg astragaloside IV from Week 5 (Figure 5b). Aortic MDA level was significantly higher in fructose-fed rats than in the normal control group. Treatment of rats with astragaloside IV from Week 5 reduced the increase in MDA level seen in fructose-fed rats (Figure 5c).

In the present study, serum insulin was increased, TG levels were elevated, glucose tolerance was impaired and blood pressure was higher in fructose-fed rats, suggesting that fructose feeding results in a model of metabolic syndrome, as reported previously [8,10,12]. The mechanisms involved in the fructose-induced metabolic disturbances and endothelium dysfunction seen in the present study may contribute to the development of obesity and accompanying insulin resistance seen following the increased consumption of dietary fructose in animals [8,12]. 

Astragaloside IV, a 3-*O*-β-D-xylopyranosyl-6-*O*-β-D-glucopyranosylcycloastragenol, was purified from the Chinese medical herb *Astragalus membranaceus* (Fisch) Bge. which has been prescribed for centuries in China as one of the most popular herbs, widely used clinically in many disorders including cardiovascular diseases. It is known that astragaloside IV can protect the myocardium and central nervous system against ischaemic injury [1,2,3,4]. It also produces vasorelaxation, mainly through the nitric oxide (NO)–cGMP pathway. Recently, it has been reported that astragaloside IV can increase insulin-induced preadipocyte differentiation, improve high glucose-induced insulin resistance in adipocytes and prevent tumour necrosis factor (TNF)-α-induced apoptosis in endothelial cells *in vitro.*

Using the fructose-fed rat as a model of metabolic syndrome, we demonstrated that chronic administration of astragaloside IV dose-dependently ameliorated the increases in serum insulin and lipid abnormalities. The highest dose of astragaloside IV tested (2 mg/kg) also prevented the increase in blood pressure seen in fructose-fed rats and improved glucose tolerance. Furthermore, endothelium-dependent vessel relaxation was significantly impaired in aortic rings from fructose-fed rats, with decreased tissue NO production and increased lipid peroxidation also seen in these rats. Treatment of fructose-fed rats with astragaloside IV resulted in an improvement in endothelium-dependent vessel relaxation and increased tissue NO and cGMP production in the myocardium and aorta.

Although the underlying pathogenesis of metabolic syndrome is not fully understood, considerable evidence now indicates that insulin resistance may be a central abnormality in the syndrome and that there is a complicated interplay between insulin resistance, abnormal lipid metabolism and endothelial dysfunction that promotes the development of metabolic syndrome. Metabolic syndrome is a leading cause of vascular injury, with increased contraction and impaired relaxation, as well as structural changes [13,14,15,16]. The endothelium is thought to play a critical role in maintaining vascular homeostasis, a process dependent on the balance between the production of NO, superoxide and other vasoactive substances. Insulin resistance may be linked to endothelial dysfunction by a number of mechanisms, including disturbances of subcellular signalling pathways common to both insulin action and NO production [17]. In the present study, we observed impaired endothelium-dependent vessel function along with decreased tissue NO production in insulin-resistant, fructose-fed rats. The improvement in endothelial function with a concomitant increase in tissue NO and cGMP levels following astragaloside IV treatment indicates that astragaloside IV may correct the major abnormalities in our model of metabolic syndrome partly via effects on the NO/cGMP pathway.

The tissue content of cGMP was increased in the aorta and myocardium of astragaloside IV-treated, fructose-fed rats. These results could be taken as an indication that the vessels in the tissues of astragaloside IV-treated, fructose-fed rats may have tendency to relax, which may be due, in part, to an augmentation of the endothelial NO/cGMP pathway, as reported previously [6]. It has been suggested that an impairment of the NO system is important in the development of hypertension in metabolic syndrome [13,14]. In the fructose-fed, insulin-resistant rats in the present study, the endothelial dysfunction may have also contributed to the higher blood pressure in addition to the hyperlipidaemia, both of which are important factors contributing to the poor cardiovascular outcomes in metabolic syndrome. Astragaloside IV ameliorated the fructose-induced changes, increasing both myocardial and aortic NO production, suggesting a beneficial role for astragaloside IV in preventing metabolic syndrome via an NO-related pathway. The fact that the lower dose of astragaloside IV used in the present study did not produce a significant improvement in endothelium-dependent vasodilation suggests that there are other ways in which endothelium dysfunction contributes to metabolic syndrome. 

Malondialdehyde levels in the left ventricle and aorta, as indicators of lipid peroxidation, were significantly higher in fructose-fed rats than in the normal control group; however, astragaloside IV treatment of fructose-fed rats decreased the fructose-induced increase in MDA concentrations. Increased inflammation and defects in the NO system may be important factors contributing to the development of metabolic syndrome [13,17,18,19,20,21]. In a previous study, we demonstrated that astragaloside IV can act as an anti-oxidant [1]. The anti-oxidant effect of astragaloside IV, along with its ability to improve NO production, may underlie its amelioration of the metabolic abnormalities seen in the fructose-fed rats in the present study.

In addition, previous studies have evaluated the gene profile after chronic intraperitoneal astragaloside IV treatment and have reported that astragaloside IV significantly upregulates genes involved in blood vessel development [22]. These findings suggest that astragaloside IV has a potential effect on vessel-related pathways to exert its effects on vessel function, hypertension and insulin resistance. Evidence supporting the involvement of other pathways in the regulation of vessel function by astragaloside IV includes the findings that astragaloside IV increases insulin-induced preadipocyte differentiation, improves high glucose-induced insulin resistance in adipocytes and prevents TNF-α-induced apoptosis in endothelial cells *in vitro* [7]. Thus, the mechanisms involved in the effects of astragaloside IV to ameliorate insulin resistance may be related to its anti-oxidant action as well as protection of endothelium-dependent vasorelaxation through the NO/cGMP pathway; these issues require further investigation.

## 3. Experimental

### 3.1. Source of Astragaloside IV

Astragaloside IV was isolated from the root of *Astragalus membranaceus* (Fisch) Bge*.*, collected at Longxi (Gansu, China) and identified by Professor Hancheng Zheng (Department of Pharmacognosy, School of Pharmacy, Second Military Medical University, Shanghai, China). A voucher specimen (no. 021003–11) has been deposited in the herbarium of the School of Pharmacy, Second Military Medical University [1]. The purity of astragaloside IV was determined to be 95.5% by HPLC analysis. Figure 1 shows the chemical structure of astragaloside IV (3-*O*-β-D-xylopyranosyl-6-*O*-β-D-glucopyranosyl-cycloastragenol, C_41_H_68_O_14_, molecular weight 784).

### 3.2. Animals and Fructose Feeding

Forty-eight male Sprague-Dawley rats (weighing 230–250 g) were housed individually in a temperature-controlled room under a 12 h dark–light cycle, with free access to standard chow. Rats were divided into four groups (n = 12 in each group): (i) normal control rats, given tap water for eight weeks; (ii) rats receiving 10% fructose in their drinking water for eight weeks (fructose-fed rats) [8,12]; (iii) fructose-fed rats treated with 0.5 mg/kg, i.p., astragaloside each day from the beginning of week 5; and (iv) fructose-fed rats treated with 2 mg/kg, i.p., astragaloside each day from the beginning of week 5. Rats in the normal control and fructose-fed control groups were injected daily with an equal volume of vehicle (dimethylsulfoxide, DMSO) from the beginning of week 5. After the 8-week treatment period, rats were subjected to an *in vivo* glucose tolerance test (GTT) and then *in vitro* experiments were performed to evaluate vessel function. All experimental procedures were approved by the Animal Care Committee of the Animal Center at the Chinese Academy of Sciences in Shanghai.

### 3.3. Blood Pressure Measurement and GTT

After rats had been fed with fructose for eight weeks, blood pressure was determined using a standard tail-cuff method with a blood pressure machine (BP-98A, Softron Beijing Incorporated). Simplely speaking, the rats are kept warm at 37 °C to optimize cardiovascular circulation within a cylindrical heater, with the exception of the tail, and further wrapped in an inner cover of cotton sheet. A programmable sensor attached to a tail (or inflatable balloon) cuff is used to monitor for tail pulse waves and to measure blood pressure when the pulse waves become stable and rhythmic. The following day, rats were subjected to a GTT: rats were injected with 2 g/kg, i.p., glucose, with blood glucose determined 15, 30, 60, 90 and 120 min after glucose loading [8,12,13].

### 3.4. Determination of Blood Lipids, Insulin, Tissue NO Metabolites and Malondialdehyde

After the GTT had been completed, rats were anaesthetized with sodium pentobarbital (60 mg/kg, i.p.) and blood was collected for the determination of: (i) serum insulin, using a rat radioimmunoassay (RIA) kit (Linco Research, St Charles, MO, USA); and (ii) cholesterol and triglyceride (TG), using an automatic analyser (Hitachi, Tokyo, Japan). Myocardial and aortic production of endogenously formed thiobarbituric acid-reactive malondialdehyde (MDA), as an index of lipid peroxidation, and nitrate and nitrite (NO_x_), NO metabolites, were determined with commercially available assay kits (Jian Cheng, Nanjing, China) according to the manufacturer’s instructions. Part of the descending aorta, with perivascular tissue and the left ventricular myocardium, was taken for the determination of tissue cGMP levels, whereas the remaining segment of the aorta was used for *in vitro* experiments (see below).

### 3.5. Preparation of Thoracic Aortic Rings and Tension Recording

Aortic segments were placed in Krebs’–Henseleit buffer (composition in mmol/L: NaCl 118.0; KCl 4.7; CaCl_2_ 2.5; MgSO_4_ 1.2; KH_2_PO_4_ 1.2; NaHCO_3_ 25.0; glucose 11.0; Na_2_-EDTA 0.5) and were then cleaned of perivascular fat and connective tissue, cut into 2–3 mm segments and mounted on two stainless steel hooks in a 10 mL organ bath containing Krebs’–Henseleit buffer gassed continuously with 95% O_2_/5% CO_2_ and maintained at 37 °C. One hook was connected to an isometric force transducer to measure tension.

Rings were stretched to a resting tension of 2.0 g and allowed to equilibrate for approximately 60 min, with the bath buffer changed every 10–15 min. Endothelium-dependent and -independent vessel relaxation was assessed qualitatively in 1 μmol/L phenylephrine-precontracted rings as the degree of relaxation caused by the cumulative addition of increasing concentrations of acetylcholine (ACh; 10 nmol/L–10 μmol/L) and sodium nitroprusside (SNP; 1 nmol/L–1 μmol/L), respectively. Furthermore, the effects of inhibition of NO synthase (NOS) were evaluated using *N*^G^-nitro-l-arginine methyl ester (l-NAME). In these experiments, rings were incubated with 10 μmol/L l-NAME for 30 min and then endothelium-dependent relaxation in response to ACh was re-examined.

### 3.6. Determination of Tissue cGMP

The aorta with perivascular tissue was weighed and homogenized in 50 mmol/L cold acetic acid and then centrifuged at 13,000 g for 10 min. The precipitate was extracted twice with ethanol for 5 min each time and the supernatant collected in a glass bottle and dried for determination of cGMP using a commercially available RIA kit (Shanghai Traditional Medicine University Medical Radiology Laboratory, Shanghai, China).

### 3.7. Statistical Analysis

Data are expressed as the mean±SEM. Differences between groups were assessed using one-way anova, followed by Student–Newman–Keuls’ post hoc analysis. Differences in blood glucose, as determined by the GTT, and concentration–response curves for isolated aortic rings were evaluated using repeated-measures anova. *P* < 0.05 was considered significant.

## 4. Conclusions

In conclusion, the results of the present study demonstrate that chronic administration of astragaloside IV to fructose-fed rats ameliorates the metabolic and cardiovascular abnormalities induced by fructose feeding, including glucose intolerance and hypertension. The anti-oxidant actions of astragaloside IV and its protection of endothelium-dependent vasorelaxation through the NO/cGMP pathway may be important factors contributing to its beneficial effects in metabolic syndrome.
